# Broadening activity of checkpoint blockade agents by intratumoral nucleoside cleavage

**DOI:** 10.1172/jci.insight.194142

**Published:** 2026-05-22

**Authors:** Regina Rab, Jeong S. Hong, Brendan L.C. Kinney, Nicole C. Schmitt, William B. Parker, Adrianna Westbrook, Kelsey B. Bennion, Mandy L. Ford, Douglas H. Weitzel, Paula L. Miliani de Marval, Eric J. Sorscher, Annette Ehrhardt

**Affiliations:** 1 Division of Pulmonary, Asthma, Cystic Fibrosis, and Sleep, Department of Pediatrics, Emory University School of Medicine and Children’s Healthcare of Atlanta, Atlanta, Georgia, USA.; 2 Department of Otolaryngology – Head and Neck Surgery, and Winship Cancer Institute, Emory University School of Medicine, Atlanta, Georgia, USA.; 3 PNP Therapeutics Inc., Birmingham, Alabama, USA.; 4 Pediatric Biostatistics Core, Department of Pediatrics, Emory University, Atlanta, Georgia, USA.; 5 Department of Surgery, Emory University School of Medicine, Atlanta, Georgia, USA.; 6 Charles River Laboratories and; 7 SpringWorks Therapeutics, Research Triangle Park, North Carolina, USA.

**Keywords:** Genetics, Oncology, Breast cancer, Cancer gene therapy, Cancer immunotherapy

## Abstract

We investigated whether destroying malignant cells and the associated tumor microenvironment (TME) by focal gene therapy would broaden immune checkpoint inhibitor (ICI) effectiveness. We show that ICI antitumor activity against syngeneic (murine) triple-negative breast cancer (TNBC) was augmented when a therapeutic transgene (purine nucleoside phosphorylase*,* referred to here as *E*. *coli* PNP) was used to cleave fludarabine (2-fluoro-arabinofuranosyl adenine) to the anticancer purine base, 2-fluoroadenine (F-Ade). We also established strong repression of anatomically distant, non-PNP-expressing tumors being treated by the same strategy. TNBC cytoreduction was associated with decreased intratumoral PD1^+^ Tregs, increased granzyme B^+^ NK cells, elevated MKI67^+^ T8 cells, and rapid immune clearance. Because F-Ade works by a mechanism that destroys quiescent neoplastic and supporting cells in the microenvironment, and since resistance to ICIs depends upon an intact TME, tumor killing by this approach offers a means to sensitize refractory malignancies to immune ablation and points to broad applicability against numerous cancer subtypes.

## Abstract 2

We describe a powerful new strategy that destroys breast cancers in mice by enhancing immunologic tumor clearance and ablating tumor-supporting cells in the microenvironment.

## Introduction

Improving antitumor effectiveness of immune checkpoint inhibitors (ICIs) represents a rapidly advancing priority in cancer research (1–6). For many malignancies, ICIs show marginal clinical benefit, and additional approaches that bolster tumor clearance by this approach are needed. Based on the well-established finding that neoantigen formation and diversity can predict ICI anticancer activity, transduction of mRNAs encoding tumor-specific antigens has been applied to melanoma, pancreatic, and other neoplasms (7–12). As the tumor microenvironment (TME) contributes markedly to T cell paralysis and tumor protective effects of checkpoint blockade, a number of additional strategies to augment ICIs are also under development (2–4, 13–15). However, knowledge remains far from complete regarding immunologic pathways most suitable for promoting ICI activity in vivo.

**Figure 1:**
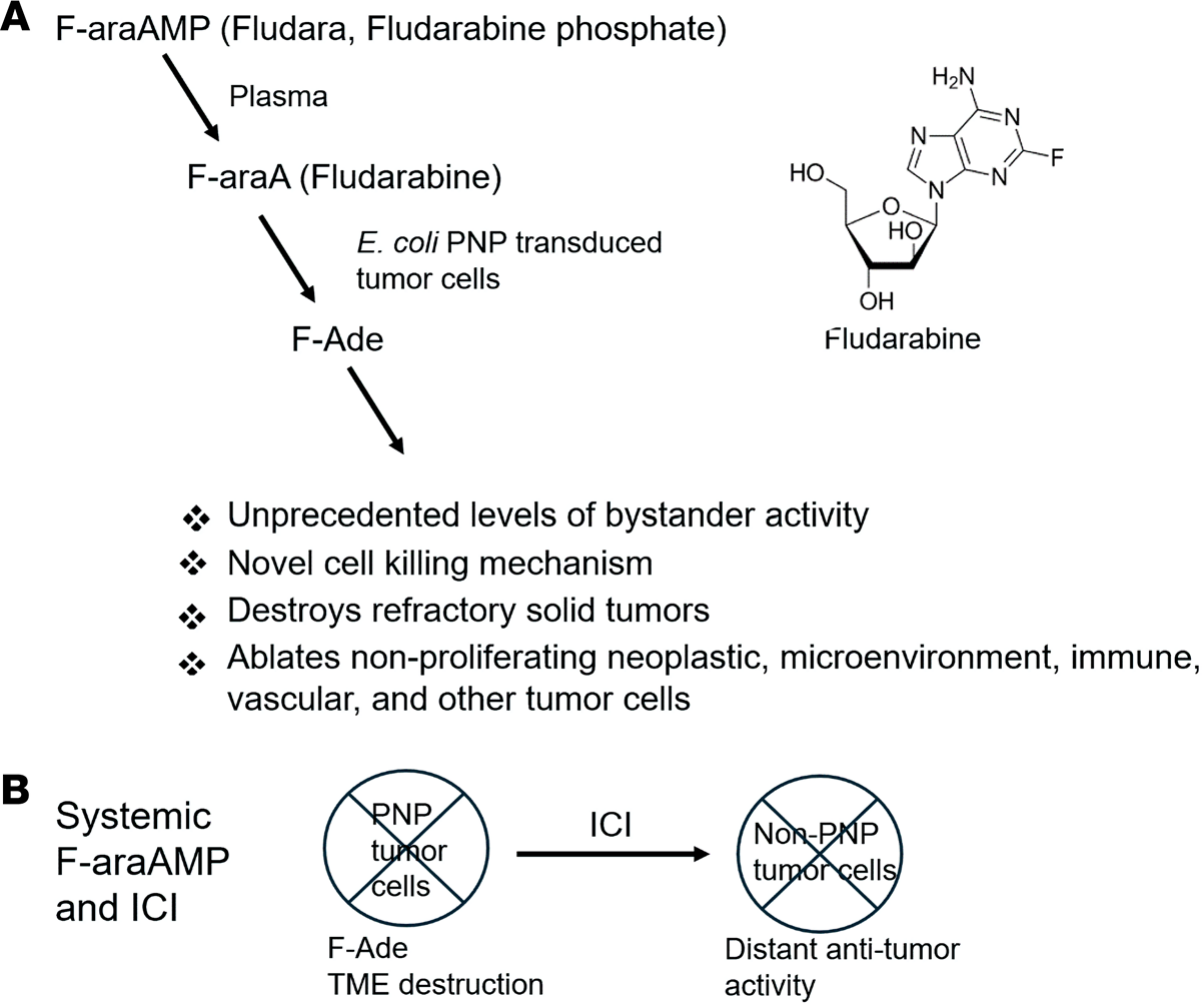
Assessment of tumor ablation with *E*. *coli* PNP and immune checkpoint inhibitors. (**A**) Prodrug activation by *E*. *coli* PNP. Fludarabine is cleaved to liberate F-Ade, a compound that disrupts DNA, RNA, and protein synthesis. (**B**) Distant tumor ablation. *E*. *coli* PNP was tested for effects on immune recognition and ICI clearance of distant EMT6 (triple-negative breast cancer) tumors not expressing the transgene. TME, tumor microenvironment; ICI, immune checkpoint inhibitor.

Intratumoral expression of the prokaryotic purine nucleoside phosphorylase (PNP) can efficiently ablate tumor tissue (Figure 1) (16–20). In this modality, 2-fluoroadenine (F-Ade) is liberated from 2-fluoro-arabinofuranosyl adenine (F-araA) specifically by bacterial (but not mammalian) PNP, which is then converted via adenine phosphoribosyltransferase to cytotoxic metabolites that disrupt DNA, RNA, and protein synthesis (17). F-Ade has remarkably high antitumor potency — orders of magnitude beyond what can be achieved with first-generation prodrug activation strategies, such as herpes simplex virus thymidine kinase or bacterial cytosine deaminase. No tumor cell types have yet been identified that are resistant to cell killing by this approach, including human head/neck, lymphoma, lung, colon, hepatic, prostate, bladder, pancreatic, and ovarian cancer as well as mouse or rat glioma and murine breast (refs. 21–38 and our unpublished observations). In vivo or in vitro, cell killing and/or obliteration of entire tumor cell populations are typically observed when a small fraction of cells (e.g., <3%–10%) express *E*. *coli* PNP (17). Moreover, because mRNA and protein synthesis are essential to malignant tissues regardless of proliferative state (i.e., whether quiescent or actively dividing), the PNP strategy provides a means to target all compartments of a tumor mass, including the TME. Conventional chemotherapy, radiation, or other anticancer modalities primarily target proliferating tumor cells and are therefore much less active against the noncycling compartments within a neoplasm (2, 17, 20). The unprecedented level of bystander cell killing achieved using PNP includes early evidence of clinical safety and efficacy against locoregional head and neck and melanoma neoplasms in humans using replication-deficient adenovirus to deliver transgene (39–41). Preclinical and clinical studies have established that the PNP/F-araA strategy is safe, as any F-Ade released into systemic circulation from a treated tumor is markedly diluted by the host blood volume and rapidly metabolized by xanthine oxidase, a ubiquitous enzyme in mammals (17).

Cancer gene therapy using prodrug activation has been severely limited by a requirement for direct inoculation into tumor tissue. Because F-Ade partitions freely between and among malignant cells and the TME, complete regressions have been routinely demonstrated in murine models of locoregional masses even when <10% of cellular targets express the transgene (17, 24). However, achieving transduction efficiency of even a few percentage points represents a substantial challenge in sizable masses such as those encountered in the clinic. Like other intratumoral transgenes, PNP expression typically occurs along needle tracks following intratumoral (IT) administration of either viral or nonviral delivery vehicles (24) (see also below). This restricted transduction may be insufficient for ablating large, local, or regional tumors encountered in clinical practice or for tumors with metastases.

In the present report, we examined relationships between PNP therapy and ICI-mediated cytoreduction. We show that focal destruction by PNP within 1 needle-accessible tumor can strongly promote systemic ICI activity. This includes activity against distant, non-PNP-expressing lesions (models of tumor metastases) that are strongly inhibited by the approach. While the activity of ICIs is diminished by many features of the TME — including fibroblasts, endothelium, myeloid cells, and pericytes (1–4) — PNP/fludarabine phosphate (PNP/ F-araAMP) effectively destroys these cells even if quiescent. One hypothesis underlying the current studies, therefore, is that an unprecedented ability of PNP to kill nonproliferating cells within a tumor mass can disrupt the TME, abrogate effects that confer ICI resistance, and stimulate cancer regressions. Accordingly, we evaluated gene transfer-based therapy of a triple-negative breast cancer (TNBC) highly resistant to standard treatments, including ICIs. First, we showed profound tumor cell killing using this approach in vitro and that TNBC destroyed by PNP exhibited features of immunogenic cell death (ICD), a molecular phenotype that has been associated with augmented ICI reactivity (42). Next, we established complete regressions and cures of TNBC following stable PNP transduction and treatment with F-araAMP. This allowed us to document both safety and TME elimination at maximal doses of the PNP-based therapy. When we applied subcurative PNP treatment (by reducing the level of F-araAMP), strong enhancement of either of 2 ICIs (anti-cytotoxic T-lymphocyte–associated protein 4 [anti-CTLA4] or anti-programmed cell death-ligand 1 [anti-PDL1]) was observed. Importantly, we also show that non-PNP tumors (models of metastases) are repressed by ICIs when locoregional lesions elsewhere in the host are inhibited by PNP. Finally, to examine mechanism of action, we characterized a focused group of immune cell infiltrates from both PNP-treated and untreated malignant masses. We observed changes in tumor-associated immune cells likely to mediate ICI activity in the setting of PNP/F-araA cancer destruction. To our knowledge, these experiments are the first to elucidate TNBC regression using the robust PNP tumor sensitization gene and provide important information regarding mechanisms that confer ICI activity in vivo against TNBC.

## Results

**Figure 2:**
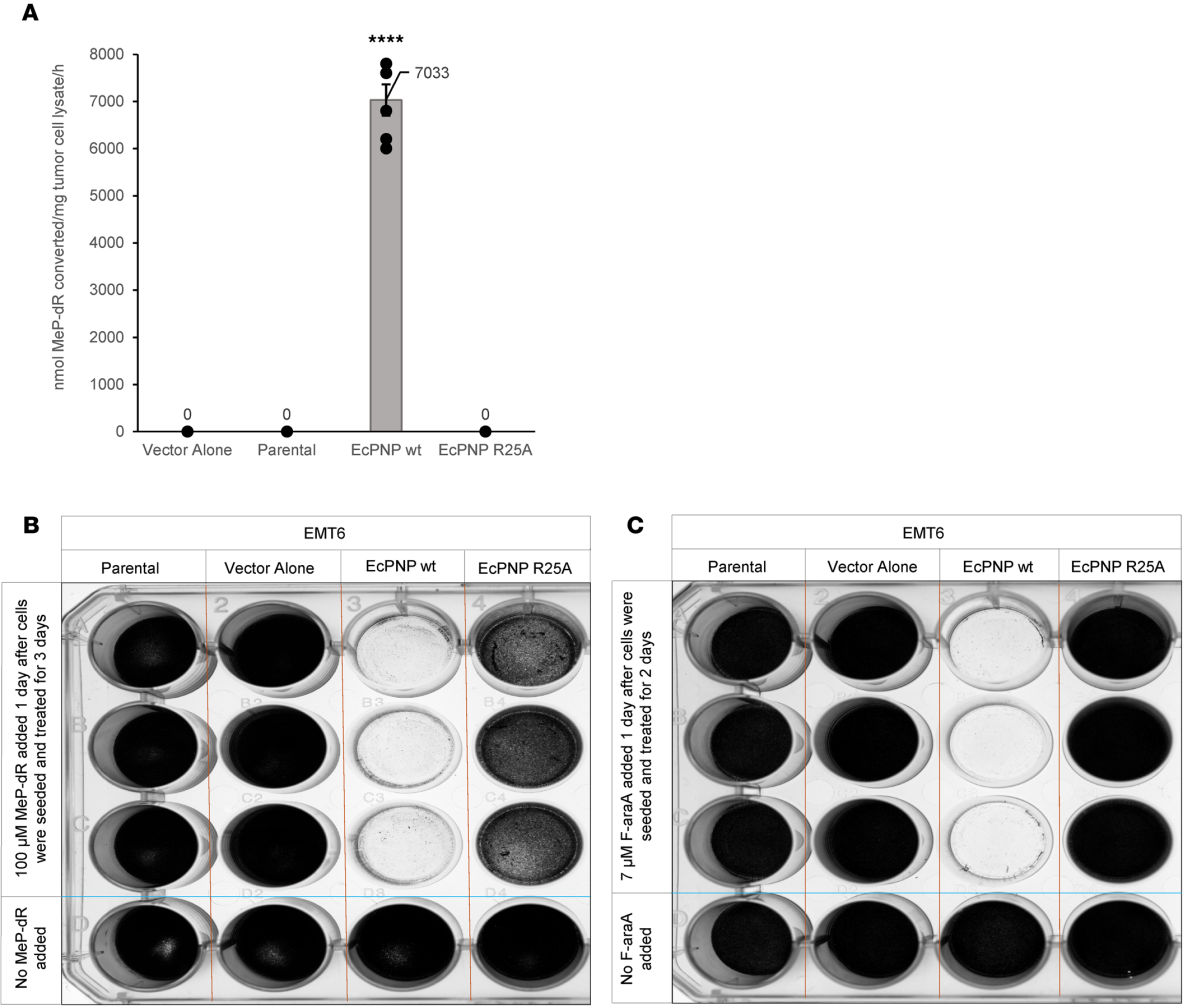
Characterization of EMT6 triple-negative breast cancer–expressing *E*. *coli* PNP. (**A**) Nucleoside cleavage activity of cell lysates (nmol prodrug converted to purine base per mg cell extract per hour; see Methods). *n* = 6 biologic replicates per condition. Data are shown as the mean ± SEM. ****P < 0.0006, 1-way ANOVA with Bonferroni’s correction for multiple comparisons. (**B** and **C**) In vitro cell killing of murine triple-negative breast cancer (TNBC) cells following cleavage of nucleoside by *E*. *coli* PNP. Cells were grown to 70%–80% confluency and treated with (**B**) 100 μM MeP-dR (F-AraA homologue used for in vitro analyses) or (**C**) 7 μM F-araA. Representative experiments are shown, with *n* = 9 biological replicates per condition from 3 independent experiments. Cells were stained with 0.1% crystal violet. Complete ablation of tumor cells in culture was observed specifically in the presence of functional *E*. *coli* PNP. Parental, TNBC without transduction; Vector Alone, TNBC transduced with empty lentiviral vector (no PNP expression); EcPNP wt, stable transduction with wild-type PNP; EcPNP R25A, TNBC transduced with *E*. *coli* PNP encoding an active site mutant with diminished activity. The HPLC assay did not report very low-level prodrug conversion responsible for barely detectable cytotoxicity shown on far right of **B**.

To test antiproliferative effects of *E*. *coli* PNP/F-araA, murine TNBC (EMT6) cells expressing *E*. *coli* PNP were generated. EMT6 was selected for these studies as a cancer model resistant to conventional treatments such as radiation, ICIs, anti–TGF-β, and other modalities (43–45). Expression of *E*. *coli* PNP was verified by HPLC to detect enzymatic function (Figure 2A) using a prototypic *E*. *coli* PNP nucleoside substrate (6-methyl purine 2’-deoxyriboside [MeP-dR]) commonly employed for in vitro analysis (17). Because MeP-dR is not metabolized by mammalian PNP, cell killing in vitro was observed specifically in the presence of functional *E*. *coli* PNP together with nucleoside (Figure 2B). Similar results were obtained with F-araA, a close analog of MeP-dR and the bioavailable form of the clinically approved compound (F-araAMP) (Figure 2C). EMT6 cells transduced with wild-type *E*. *coli* PNP showed enzyme activity in a range known to mediate strong antitumor effects in vivo (17, 24).

**Figure 3:**
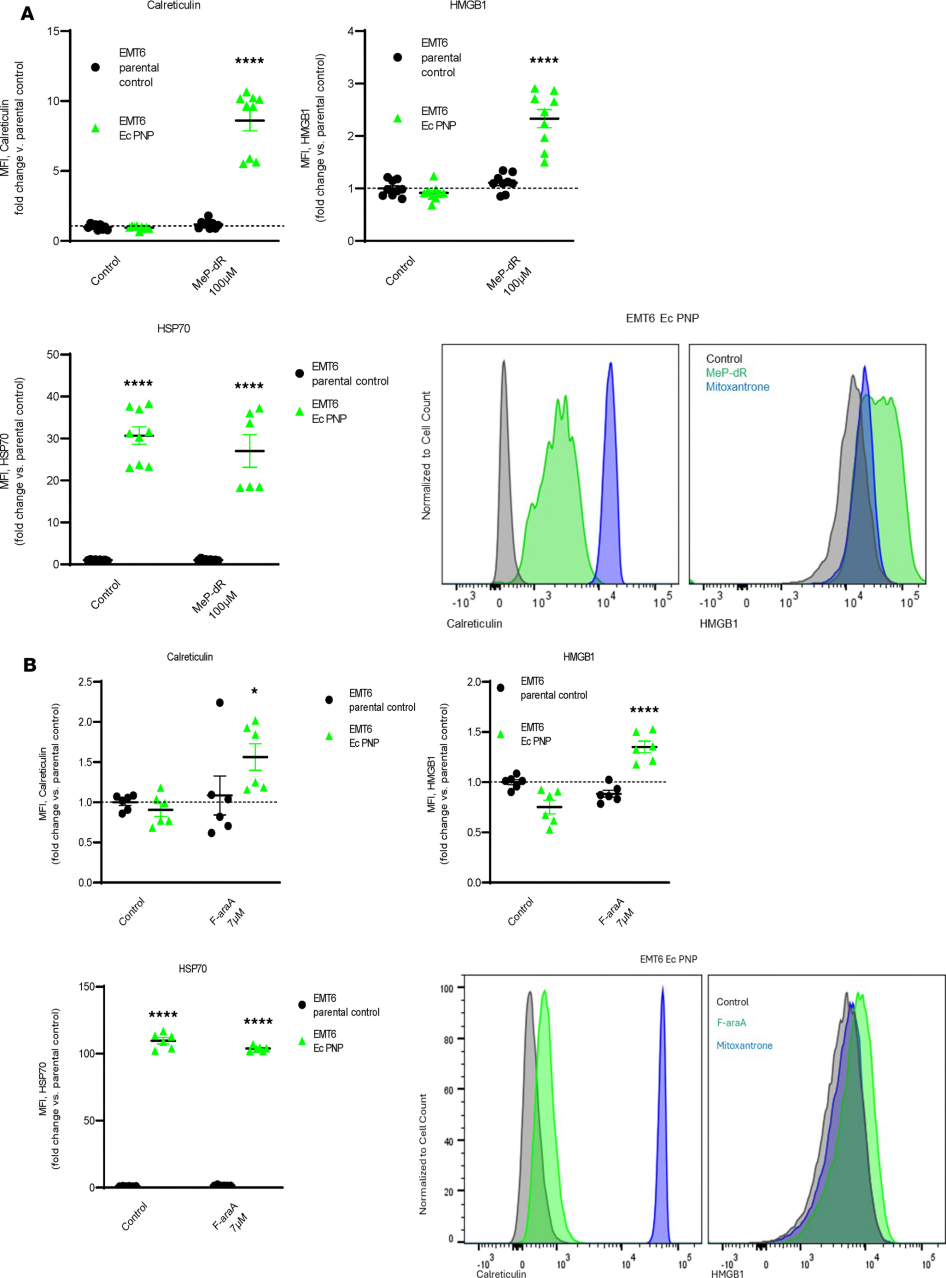
Nucleoside prodrug treatment of *E*. *coli* PNP–expressing EMT6 cells elicits signaling pathways associated with immunogenic cell death. (**A** and **B**) Cells were treated as in Figure 2 with *E*. *coli* PNP substrate (MeP-dR; **A**) or fludarabine (F-araA; **B**) for 24 hours, then fixed, stained for immunogenic cell death (ICD) markers, and analyzed by flow cytometry. *P < 0.05; ****P < 0.0001, versus parental control, 2-way ANOVA (post hoc Tukey’s comparison). Data shown as the mean ± SEM; *n* = 6–9 biologic replicates combined from 2–3 independent experiments performed in triplicate. Flow cytometry histograms are shown for each experiment following PNP/nucleoside treatment or treatment with mitoxantrone (1 μg/ml × 24 hours), a known ICD inducer, as positive control. Cells expressing *E*. *coli* PNP exhibit basal elevations of HSP70 at the plasma membrane. When EMT6 cells expressed the R25A (inactive) PNP enzyme, ICD response was minimal (Supplemental Figure 1). Ec PNP, wild-type *E*. *coli* PNP; MFI, mean fluorescence intensity; CALR, calreticulin.

ICD is a well-described process by which clearance of a malignant mass can be promoted. Specific damage-associated molecular profiles (DAMPs) are used to track ICD responsiveness. Characteristics of the DAMP phenotype include cell surface–localized calreticulin and heat shock protein HSP70 (HSPA1A), together with cellular production and release of HMGB1 — contributing to inflammation and immune activation (42). EMT6 cells expressing *E*. *coli* PNP were treated with MeP-dR and analyzed for DAMPs by flow cytometry (Figure 3A), under conditions that exhibit robust cell killing (Figure 2B). Surface expression of calreticulin was strongly elevated compared with that of untreated tumor cells (*P* < 0.0001; Figure 3A). Abundance of HSP70 was also augmented following recombinant PNP expression (*P* < 0.0001). After 24 hours, increased HMGB1 (*P* < 0.0001), likely corresponding to repackaging of intracellular vesicles prior to release (46, 47), was observed. Similar results were noted for F-araA as an *E*. *coli* PNP substrate (Figure 3B). The DAMP phenotype was not effectively induced when an inactive R25A *E*. *coli* PNP was examined (Supplemental Figure 1; supplemental material available online with this article; https://doi.org/10.1172/jci.insight.194142DS1). Features of wild-type PNP protein biogenesis or function therefore appear necessary to establish a more complete DAMP. In either case, the findings show that *E*. *coli* PNP–based treatment triggers a signaling cascade that has previously been associated with immune sensitization and tumor reduction by ICIs.

**Figure 4:**
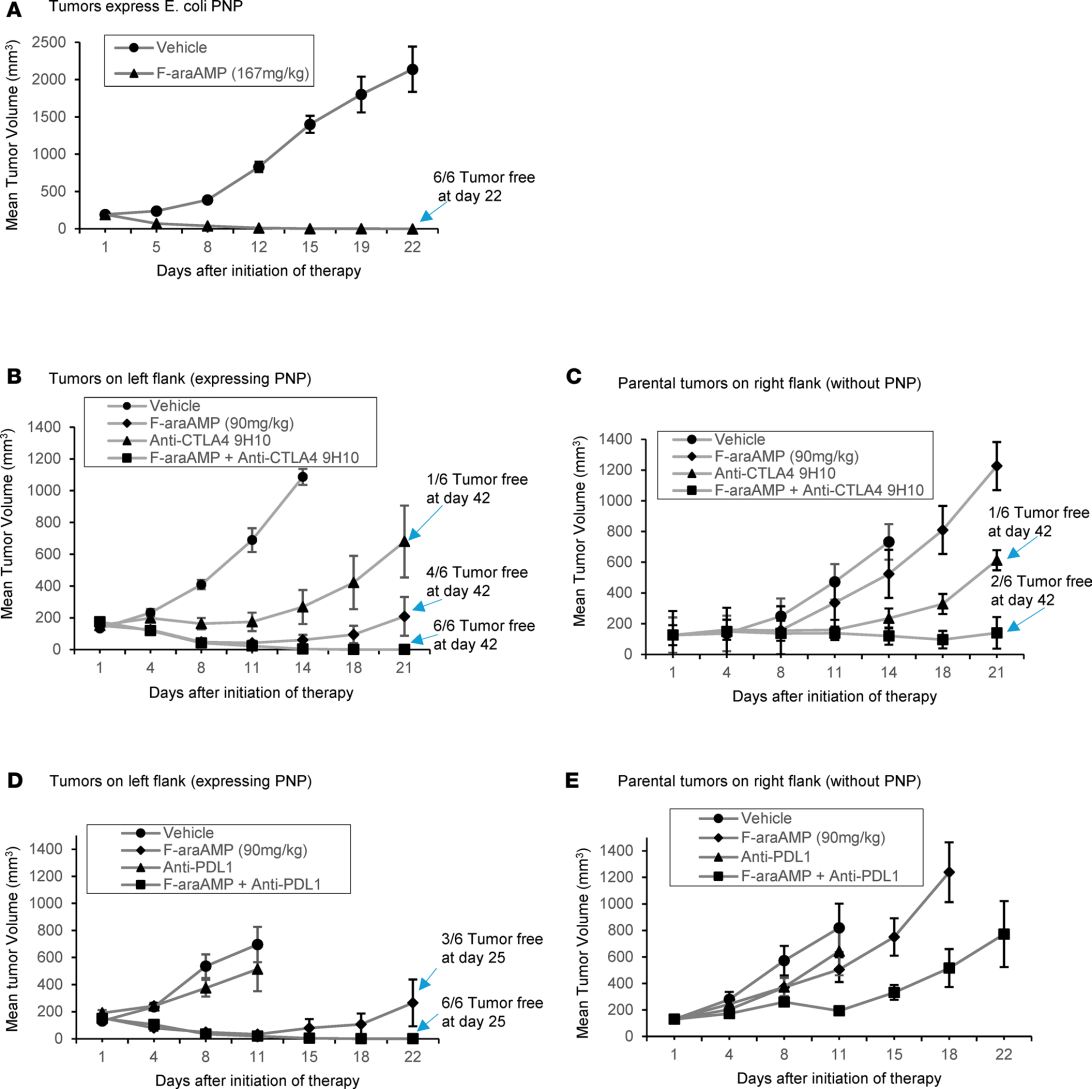
Combining immune checkpoint inhibition with PNP/F-araAMP augments ICI activity against triple-negative breast cancer. (**A**) F-araAMP was administered i.p. at a dose of 167 mg/kg on days 1–3 after tumors reached a size of 100–200 mm^3^. (**B**–**E**) Mice were implanted with bilateral tumors expressing PNP (in the left flank, **B** and **D**) or no PNP (right flank, **C** and **E**). F-araAMP was administered (by intent) at subcurative dosing (90 mg/kg i.p. on days 1, 2, and 3). (**B** and **C**) Anti–CTLA-4 was given i.p. on days 1, 4, and 7 (5 mg/kg on day 1 and 2.5 mg/kg on days 4 and 7). Anti-PDL1 (**D** and **E**) was dosed i.p. biweekly for 2 weeks (days 1, 4, 8, and 11). *E*. *coli* PNP activity in the EMT6-transduced line was 6,000–7,000 units, with zero activity in parental tumors not expressing PNP. Statistical analysis of tumor volume was performed on day 21 for **B** and **C** and days 15 and 18 for **D** and **E**. The ICI cohort was found to have significantly larger tumor volumes compared with combined (ICI + F-araAMP) treatment for experiments in **B** and **C** (*P* < 0.05) and **D** (*P* < 0.025). In **E**, because animals were removed/euthanized owing to rapid tumor growth, an alternative analysis for tumors that doubled in size by day 11 was performed, showing that ICI + F-araAMP differed from ICI alone (*P* < 0.05, 2-tailed *t* test with Bonferroni’s correction) (*n* = 5–6 animals per condition). Animals tolerated interventions with minimal weight loss. Data are shown as the mean ± SEM.

Antitumor activity of *E*. *coli* PNP was next evaluated in syngeneic (BALB/c) mice harboring TNBC (Figure 4). Tumors with stable *E*. *coli* PNP expression were grown to a size of 150–200 mm^3^. In vivo treatment with F-araAMP (167 mg/kg/d i.p., 3 times per day for 3 days), a clinically approved compound that is rapidly converted by mammalian plasma to F-araA, led to complete regressions and cures of PNP-expressing masses in all animals (Figure 4A). EMT6 parental (non-PNP-expressing) tumors exhibited a modest anticancer effect after treatment with saturating concentrations of anti-CTLA4 antibody (Figure 4C). Anti-PDL1 antibody alone also had little effect on parental tumors (Figure 4E). To test enhancement of ICIs using PNP, we investigated subcurative doses of F-araAMP (90 mg/kg/d, 3 times per day for 3 days). Pronounced augmentation was observed following treatment with checkpoint blockade agents in combination with PNP/F-araAMP (Figure 4, B and D), as indicated by complete regression of tumors in treated animals. Note that growth of parental EMT tumors was not altered by fludarabine without PNP-mediated nucleoside cleavage (Figure 4, C and E, and Supplemental Figure 2). Importantly, ICI blockade of non-PNP-expressing parental tumors was enhanced when a PNP-expressing mass treated with F-araAMP regressed contralaterally in the same host (Figure 4, C and E). This effect on distant (non-PNP-expressing) tumors (Figure 4, C and E) was attributable to a single cycle (days 1–3) of PNP/F-araAMP.

As additional controls, non-PNP-expressing (parental) TNBC tumors were established bilaterally and treated using the same regimen shown in Figure 4, B and C. No effect on tumor growth was noted under these conditions without PNP, although ICI sensitivity was marginally improved by F-araAMP (Supplemental Figure 3; which differs modestly from ICI activity when PNP is present, compared with Figure 4, B and C, *P* = 0.017). Our results therefore demonstrate strong augmentation of ICIs in tumors otherwise resistant to immune modulation. Moreover, non-PNP-expressing neoplasms were strongly sensitized to ICIs by the PNP-based gene transfer intervention described here.

### PNP/F-araAMP treatment results in a less immunosuppressive TME.

**Figure 5:**
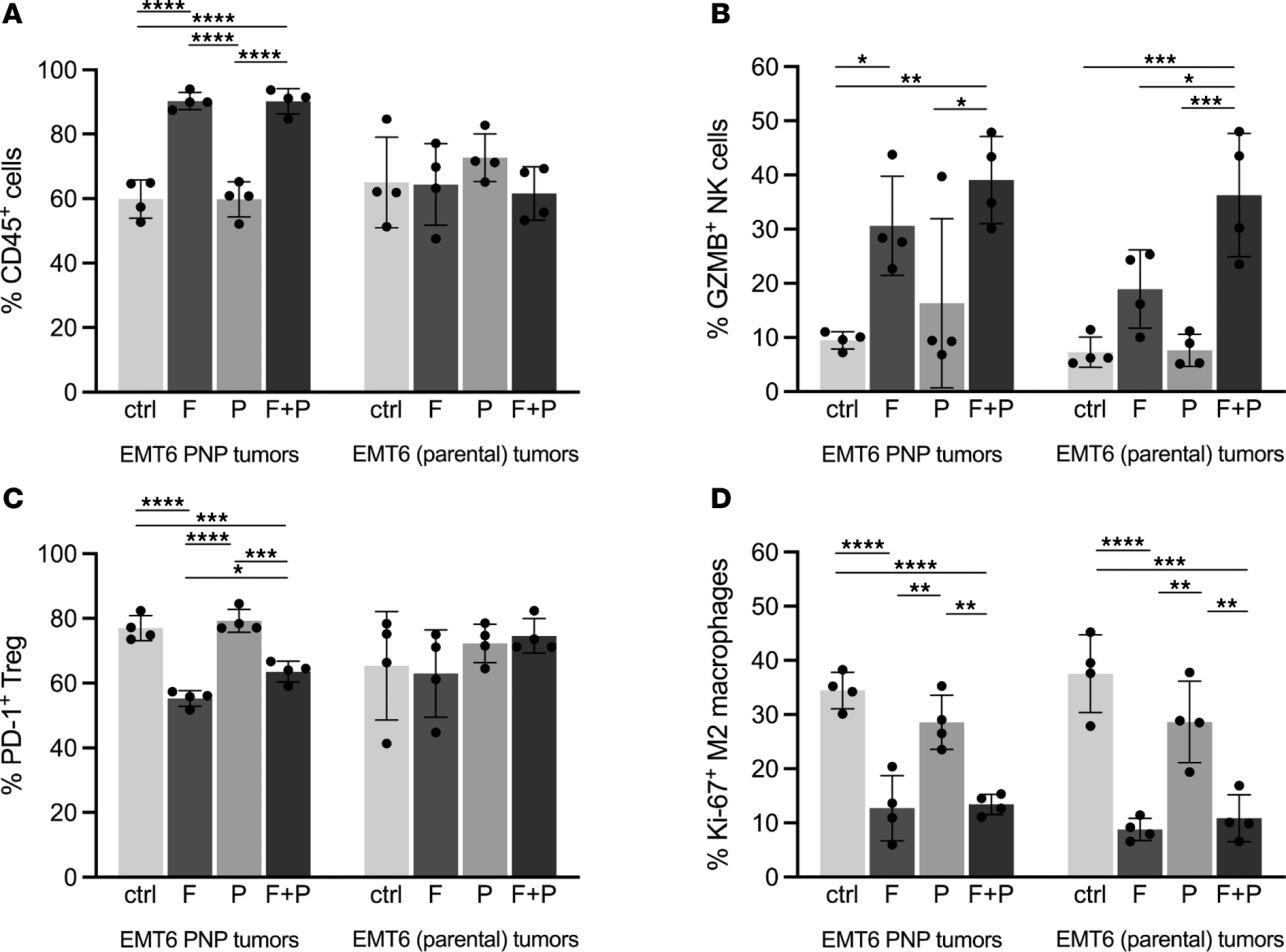
PNP/F-araAMP treatment is correlated with a less immunosuppressive tumor microenvironment. Tumors were established as in Figure 4. Parental EMT6 cells, or EMT6-cells expressing PNP, were injected into the right and left flanks of each mouse, respectively, at 5 × 10^6^ cells per flank. Injections with fludarabine phosphate (F), anti-PDL1 (P), a combination of F-araAMP and anti-PDL1 (F+P), or vehicle control (ctrl) began on day seven after implantation. Tissues were harvested for analysis of immune cells on day 5 after start of treatment. *P < 0.05; **P < 0.01; ***P < 0.0005; ****P < 0.0001. The total or percentage of CD4 and CD8 cells did not differ between any of the treatment groups except for a modest increase of the percentage of CD4-positive cells in the fludarabine versus anti-PDL1 treatment cohorts (*P* = 0.03) and an increased percentage of Ki67^+^ (MKI67^+^) CD8 cells (Supplemental Figure 5) in tumors treated with PNP/fludarabine (indicating measurable CD8 T cell activation). One-way ANOVA was used with Tukey’s multiple comparison tests for all analyses. Percentages of cells in the immune subset are shown: (**A**) percentage of all tumor cells; (**B**) percentage of NK cells; (**C**) percentage of Tregs; (**D**) percentage of M2 macrophages. Data are shown as the mean ± SD.

To begin assessment of changes in immune cell proportions within the TNBC microenvironment, tumor-infiltrating cells were investigated by flow cytometry using markers for T cell subsets (CD4, CD8, Tregs), NK cells, monocytic and polymorphonuclear myeloid-derived suppressor cells, and M1-like or M2-like macrophages. Tumors were treated similarly to those shown in Figure 4 using subcurative doses of F-araAMP and analyzed at day 5 after drug therapy (i.e., prior to complete regression/TME dissolution and following 2 doses of anti-PDL1 antibody). Percentages of CD45^+^ cells were increased specifically in PNP tumors given F-araAMP, indicating leukocyte infiltration associated with cytoreduction (Figure 5A). Comparable increase was not observed in fludarabine-treated parental tumors (Figure 5A). Total NK cells were enriched in PNP tumors following either F-araAMP or anti-PDL1 plus F-araAMP treatment compared with controls (*P* < 0.02) (data not shown). A strong increase in frequency of granzyme B^+^ NK cells in all tumors treated with PNP/F-araAMP (including distant [non-PNP-expressing] masses) was also observed (Figure 5B).

In addition to change in NK cell activation status, alterations in immunosuppressive subsets within treated tumors were noted. The frequency of PD1^+^ Tregs was found to be decreased specifically in TNBC expressing *E*. *coli* PNP after treatment with either F-araAMP or F-araAMP plus anti-PDL1 (Figure 5C). Diminished PD1^+^ Tregs were not observed in contralateral (non-PNP-expressing) tumors in the same animals. In addition, diminished frequency of MKI67^+^ tumor-infiltrating M2-like macrophages was noted in cancers treated with either F-araAMP or F-araAMP plus anti-PDL1 (including distant [non-PNP-expressing] masses; Figure 5D). These data indicate that combination treatment using ICIs with PNP/F-araAMP may promote a less immunosuppressive TME (see also below).

## Discussion

Results from the present study describe advances relevant to augmenting ICI effectiveness in vivo. First, we demonstrated that for tumors incompletely treated by ICIs, focal gene transfer using PNP/F-araAMP could markedly enhance ICI regressions (Figure 4, B and D). Combination treatment of PNP with ICIs led to strong antitumor activity and cures of locally invasive masses. Second, we furnish evidence that a PNP-based approach could also sensitize distant lesions to ICIs (Figure 4, C and E). Our findings suggest that, in patients with TNBC, IT treatment of a superficial mass with PNP/F-araAMP followed by systemic ICIs could provide a means to effectively cytoreduce both the target lesion and metastatic tumors otherwise poorly addressed by available anticancer agents.

The PNP technology described here ablates both proliferating and nonproliferating cells within tumor parenchyma, including neovasculature and TME components that influence immune surveillance (2, 4, 17). The underlying mechanism (disruption of DNA, RNA, and protein synthesis by F-Ade) is distinct among both approved and experimental anticancer strategies (17, 48). Most conventional chemotherapies or radiotherapies — and early-generation prodrug activation genes such as herpes simplex virus thymidine kinase or bacterial cytosine deaminase — interrupt DNA synthesis and are largely ineffective against nonproliferating neoplastic or TME cells (1, 4, 17, 18). In contrast, F-Ade targets both quiescent and actively cycling tumor parenchyma and is intended to abolish malignancies that escape conventional treatments because of low growth fraction. Disruption of tumor cells and the TME by PNP/F-araAMP leads to cures of aggressive masses in vivo (17) (Figure 4A). Safety of the approach has been demonstrated in preclinical (17) as well as clinical (39–41) studies. Any F-Ade that escapes from a tumor into the systemic circulation is rapidly metabolized by xanthine oxidase, a widely expressed enzyme in mammalian tissues.

We provide data indicating that (a) F-Ade produced by cleavage of F-araA destroys EMT6 tumor cells in vitro (Figure 2C), (b) F-Ade–dependent killing of EMT6 cells leads to a DAMP phenotype associated with increased ICI sensitivity (Figure 3 and Supplemental Figure 1) (42), and (c) F-araA (without conversion to F-Ade) has negligible anticancer activity in vitro or in vivo (Figure 2C, Figure 4, and Supplemental Figures 2 and 3). In previous studies, we have shown that direct IT inoculation of F-Ade results in leakage of the drug from malignant tissue and minimal antitumor effect (unlike the pronounced cytoreduction observed when F-Ade is generated intracellularly and near molecular targets) (31). In vivo studies of IT F-Ade have therefore been difficult to interpret. Use of PNP to enhance ICIs represents an important means of extending the prodrug activation strategy described here from locoregional antitumor therapy to an approach much more suitable for treating distant metastases.

The TME plays an essential role during tumor resistance to ICIs (2). Immunoregulatory pathways that govern CD8^+^ and CD4^+^ T cell activity, cytokine release, growth factors, and checkpoint signaling are all orchestrated by the TME and represent barriers to ICI effectiveness (2). While previous approaches to enhance ICIs have focused on combined treatments with chemo- or radiotherapy, broad spectrum immune adjuvants, or other methods (1, 3, 4, 49, 50), use of gene therapy to focally abolish both malignant cells and supporting TME have not been adequately investigated. Our findings establish that applying F-Ade to destroy both dividing and nondividing cells can markedly improve antitumor activity against TNBC with ICIs. Moreover, immune enhancement at one tumor site — with exposure of neoantigens and other tumor constituents — promotes a surprising level of ICI activity against distant cancers. The current report therefore furnishes an important means by which “immune antitumor paralysis” might be overcome in both local and metastatic malignant lesions. Future studies in other syngeneic murine tumor types will be needed to evaluate spectrum of activity for the present findings both therapeutically and mechanistically.

Previous preclinical and clinical studies have suggested that immunologic clearance of refractory tumors can be augmented by *E*. *coli* PNP/F-araAMP, even in the absence of ICIs. For example, Martiniello-Wilks et al. showed that treatment of aggressive RM1 murine prostate cancers with ovine adenovirus encoding *E*. *coli* PNP, followed by F-araAMP administration, inhibited growth of both primary tumors and non-PNP-transduced pseudometastases (27). Moreover, in a phase I clinical study of PNP/F-araAMP directed toward needle accessible head and neck, adenoid cystic, or melanoma tumors, PNP conferred anticancer activity despite the expectation that only small numbers of parenchymal cells were productively transduced. In that study, certain tumors without PNP exhibited modest regression when a mass elsewhere in the patient was being inhibited owing to local generation of F-Ade. These clinical results are compatible with cell signaling pathways activated using PNP in vitro (Figure 3) as well as immune cellular infiltrates observed following treatment, with or without ICI coadministration (Figure 5).

Malignant tissue destruction by PNP/F-araAMP not only abolishes the TME (Figure 4A) but would be expected to expose and release a diverse array of neoantigens, many of which are expressed in nondividing cells. We suggest that tumor-specific antigen presentation may be promoted by local treatment as described here. While additional studies will be required to formally test that assertion, our results are consistent with a body of recent data indicating the importance of the TME, together with T cell exhaustion, as contributors to ICI resistance (2, 3, 51, 52). As one example, because granzyme B is an established contributor to NK tumor cell lysis and killing (53–58), the finding of increased granzyme B^+^ NK cells and total NK cells in murine tumors treated with PNP/F-araAMP plus ICIs suggests NK cells contribute during destruction of both PNP-expressing tumors and contralateral (non-PNP-expressing) lesions. Furthermore, a diminished frequency of PD1^+^ Tregs in tumors following combination therapy was observed. Since PD1^+^ Tregs have recently been shown to negatively affect efficacy of anti-PD1 therapy (59), and since PD1^+^ Tregs are thought to be more immunosuppressive than PD1^–^ Tregs (60), the observation that PNP/F-araAMP plus ICI therapy decreases PD1^+^ Tregs in the TME is likely to be of mechanistic importance. F-araAMP– and ICI-treated mice also exhibited lower frequencies of proliferating (MKI67^+^) M2-like macrophages. The M2-like macrophage cellular compartment is known to inhibit immune responsiveness against tumor tissues (61, 62). Similar decreases were not observed when MKI67^+^ M1-like macrophages were examined (Supplemental Figure 4), a result supporting specificity of the PNP-based approach described here.

Detailed intratumoral dendritic cell assessments (numbers, subsets, spatial organization, relationship to fludarabine dosing, etc.) were not performed as part of the current analysis. While dendritic markers can be associated with ICD, numbers of these cells, per se, may represent a less consistent surrogate for checkpoint blockade inhibitor activity (63), particularly when a cancer treatment is directed toward killing cells in the TME. While the present experiments have pursued a focused set of cellular infiltrates, the PNP/ICI system developed in this report will provide an experimental tool for investigating shared immune clearance mechanisms across a variety of tumor types in the future.

Our findings establish that fludarabine by itself had no effect on EMT6 tumor growth, did not confer a DAMP-type cellular response, and only modestly augmented ICI activity in vivo. These results sharply contrast the effect when fludarabine was used to treat PNP-expressing tumors (compare Figure 4, B and C, to Supplemental Figure 3). Taken together, the data support an underlying immune mechanism during PNP tumor regression that warrants subsequent evaluation in mice and patients. Our results are consistent with a growing body of recent data indicating importance of the TME as a contributor to ICI resistance (2, 3, 51, 52). From that perspective, findings presented here identify specific immune cell types that may represent actionable cell targets and/or correlative endpoints to predict clinical benefit. As one example, increased intratumoral granzyme B^+^ NK cells are strongly associated with enhanced PNP/F-araAMP activity — and might serve as useful surrogates for tracking breast tumor regression as part of future clinical trials.

Patient-oriented studies using recombinant adenovirus to deliver PNP transgene have indicated clinical efficacy (41), although a practical barrier involves inadequate levels of tumor transduction in vivo. For larger masses, such as those often encountered in the clinic, achieving sufficient levels of expression with viral vectors has been problematic (1, 64–67). That issue is mitigated to some extent by unprecedented levels of bystander killing conferred by *E*. *coli* PNP/F-araAMP (strong tumor regressions and cures when <3%–10% of tumor cells express the transgene) (17, 24, 38). However, even PNP expression in approximately 3% of target cells for large tumors in patients with breast cancer represents a difficult challenge.

Recent findings relevant to in vivo gene transfer involve discovery of lipid nanoparticles (LNPs) capable of transducing over 60% of cells within a growing tumor mass following IT inoculation. The approach applies barcoded LNPs, representing a large portion of available chemical space to identify the most active vectors, and confers cytoreduction of head and neck squamous cell carcinoma when combined with PNP/F-araAMP in animal models (20). A similar strategy has been applied to systemically administered LNPs that addresses metastatic lesions (68). Although comparable protocols have not yet been tested for TNBC, the LNP technology establishes that very high transduction efficiencies are now possible for targeted delivery of PNP to malignant tissues in vivo. Future studies of this sort represent an important direction for development of PNP/F-araAMP together with ICIs.

In summary, the present report illustrates (a) a robust gene transfer-based approach for expanding ICI spectrum of activity to include otherwise refractory tumors such as TNBC, (b) a means of local tumor cell control by PNP/F-araAMP that can also address metastatic lesions, (c) drug administration by an IT route to destroy the TME, and (d) improved understanding of immunologic mechanisms that underlie effectiveness of PNP/F-araAMP in combination with ICIs. Our findings suggest ways in which highly efficient gene delivery vehicles might be combined with ICIs to enhance an experimental treatment already showing promise in the clinic (40, 41). The present findings set the stage for studies to test ICI sensitization in other tumor types using immune modulation — together with the antitumor activity of PNP/F-araAMP.

## Methods

### Sex as a biological variable.

Because EMT6 is a triple negative-breast cancer model, and since malignancies of the breast are much more common in human females, congenic BALB/c female mice are typically used in studies such as those described here. This convention applies to studies of EMT6 and was employed in the current experiments. Findings in female animals with breast cancer are often applicable to breast tumors in males as well, although will this require formal evaluation.

### Generation of cell lines expressing PNP.

*E*. *coli PNP* cDNA was engineered in recombinant retrovirus using methods described previously (69). A catalytically defective (mutant) enzyme was generated by site-directed replacement of arginine 25 (with alanine) (70) and verified by DNA sequencing. Wild-type or mutant *PNP* cDNAs were cloned into the retroviral vector pMX-pie (69). This construct contains an IRES-EGFP element and puromycin resistance cassette. Retroviral supernatants were obtained from BOSC23 cells (provided by JW Schrader’s laboratory, University of British Columbia, Vancouver, British Columbia, Canada) transfected with pMX-pie plasmids, followed by transfer onto EMT6 cells (Charles River Labs). Infected lines were grown for 2–3 days and then selected with puromycin. Bulk populations of stably transduced cells were tested in vitro and in vivo.

### PNP enzymatic activity.

Crude extracts were prepared by sonication of cell pellets in 10 mM HEPES buffer (pH 7.4) containing 1 mM dithiothreitol, centrifuged at 20,000*g*, and supernatants dialyzed against 100 mM HEPES (pH 7.4) containing 20% glycerol and 1 mM dithiothreitol. Extracts were incubated at 25°C in 1,000 μL volumes containing 50 mM potassium phosphate, 100 mM HEPES (pH 7.4), and 100 μM of the prototypic PNP nucleoside substrate MeP-dR (9-(2-deoxy-β-D-ribofuranosyl)-6-methylpurine) (AstaTech) at a concentration of lysate that resulted in a linear increase of product formation during the incubation period. Reactions were stopped by boiling, and precipitated proteins removed by filtration (0.2 μm). Formation of purine base product (6-methylpurine [MeP], an F-Ade analog) was monitored using reverse-phase HPLC. Activity was expressed as PNP units (17). One unit represents 1 nmol of MeP-dR–converted per mg tumor cell extract per hour (71).

### In vitro cell killing assay.

EMT6 lines were seeded (1 × 10^5^ cells/well) on 24-well plates, and MeP-dR (100 μM) or F-araA (7 μM) was added at 24 hours after plating. Cells were monitored for 2–3 days and stained with 0.1% crystal violet to evaluate cell survival. During the assay, dead or detached cells were washed away, while attached (live) cells were stained for viability. Clear wells indicated >95% cell killing.

### Evaluation for ICD.

DAMPs (42) were monitored following treatment with PNP/MeP-dR, PNP/F-araA, mutant PNP R25A/F-araA, or vehicle, and cells harvested using trypsin/EDTA, rinsed in PBS, fixed, and permeabilized using a fixation and permeabilization kit (eBioScience; Intracellular Fixation and Permeabilization Buffer Set, catalog 88-8824-00). Staining was with antibodies against calreticulin (Abcam, ab196159), HSP70 (HSPA1A; BioLegend, 648003), or HMGB1 (BioLegend, 651403). Samples were analyzed on a BD FACSymphony A1 cytometer and further evaluated using FlowJo software (Becton Dickinson). Live cells were gated based on negative staining for FVS575V or FVS780 viability dye (BD Biosciences).

### EMT tumor growth and treatment.

Parental and *E*. *coli* PNP–expressing EMT6 tumor cells (5 × 10^6^ cells in 0.1 mL) were injected subcutaneously into opposite flanks of 8- to 12-week-old female BALB/c mice (Charles River Labs) with parental cells on the right flank and PNP cells on the left flank. Tumors were measured with calipers, and an estimate of volume was calculated using the equation (length × width^2^)/2 = mm^3^. Mice were monitored daily, and body weights and tumor dimensions were recorded. Treatments with F-araAMP or F-araAMP plus ICI were performed as described in Results when tumors were 100–200 mm^3^. Tumor growth delay (T-C) was determined as the difference in median days to 2 doublings between drug-treated and vehicle-treated groups. In some tumor protocols, parental EMT6 cells were used unilaterally or bilaterally. F-araAMP was obtained from Charles River Discovery. F-Ade was purchased from General Intermediates of Canada Inc. Anti-CTLA4 and anti-PDL1 antibodies were sourced from Bio X Cell (BEO131 and BEO101, respectively).

### Flow cytometry analysis of PNP- and ICI-treated tumors.

Mouse tumor samples were dissociated according to manufacturer’s instructions using the gentleMACS Tumor Dissociation Kit (Miltenyi Biotec). Samples were filtered through a 70-micron cell strainer and rinsed twice in PBS/2.5% FBS to remove enzymatic buffer. Single-cell suspensions (e.g., 1 × 10^6^ cells per condition) were transferred to 96-well U bottom plates. Cells were centrifuged for 5 minutes at 400*g* and resuspended in 100 μL PBS containing viability dye for 15 minutes at room temperature, protected from light. Cells were then washed with FACS staining buffer and treated with surface antibody solution. After incubation for 15 minutes at 4°C, protected from light, and 2 washing steps, cells were resuspended in 115 μL staining buffer followed by 115 μL of 1:3 diluted counting beads to a final volume of 300 μL. Plate fluorescence was acquired using a BD LSR-II Fortessa flow cytometer. Antibody sources for flow studies were anti-MKI67 (also termed anti-Ki67, Becton Dickinson, catalog 563757), anti–granzyme B (Becton Dickinson, 560213), and anti-CD45 (Becton Dickinson, 564279).

### Statistics.

Comparisons of PNP enzymatic activity in vitro were performed by 1-way ANOVA with Bonferroni’s correction for multiple comparisons. Two-way ANOVA was used to compare flow cytometry results from dispersed tumor cells as part of the ICD evaluation (with analysis followed by Tukey’s multiple comparison tests). Statistical analyses for ICD measurements were plotted using GraphPad prism 10.0.3 and/or JMP 17. For studies of tumor growth, 2-tailed 2-sample *t* tests were conducted comparing experimental groups. ICI alone was compared with the F-araAMP plus ICI group. A *P* value of less than 0.05 was considered significant for analyses conducted on only 1 time point, with *P* values of less than 0.025 considered significant for analyses examining 2 days of measurement, and Bonferroni’s correction was used for multiple comparisons. Tumor growth assessment was conducted in SAS 9.4 (SAS Institute Inc.). For experiments examining immune cellular infiltrates in tumor tissue, 1-way ANOVA with Tukey’s multiple comparisons correction was used to evaluate dispersed tumor cells. A *P* value of less than 0.05 in the latter studies was considered significant.

### Study approval.

All animal studies were conducted at Charles River Labs after review and approval from the Charles River Laboratories Animal Use Committee.

### Data availability.

All primary data described in the manuscript are available on request from the corresponding author (EJS). An Excel spreadsheet has been provided with values for data points shown in graphs and data contributing to reported means (see Supporting Data Values file).

## Author contributions

Design of research studies was performed by RR, JSH, BLCK, NCS, WBP, KBB, MLF, DHW, PLMDM, EJS, and AE. Experiments were conducted by RR, BLCK, DHW, PLMDM, and AE. Data were acquired by RR, BLCK, DHW, PLMDM, EJS, and AE and analyzed by RR, JSH, AW, BLCK, NCS, WBP, KBB, MLF, DHW, PLMDM, EJS, and AE. JSH, BLCK, NCS, DHW, PLMDM, EJS, and AE provided reagents, and the manuscript was written by RR, JSH, BLCK, NCS, WBP, KBB, MLF, DHW, AW, PLMDM, EJS, and AE.

## Conflict of interest

EJS and WBP have ownership interests in PNP Therapeutics Inc. and serve on the board of directors for the company, which develops products used in research described by the paper. JSH also has minor equity interest in the company. The terms of this arrangement for EJS and JSH have been evaluated and approved by Emory University in accordance with its conflict-of-interest policies. EJS, JSH, RR, and AE are listed as inventors on US patent applications 19/113,702 and 17/908,465. US patents 12/453,760, 10/080,784, and 8/628,767 list EJS and WBP as inventors. EJS also receives funding to his laboratory from GeoVax Labs.

## Funding support

This work is the result of NIH funding, in whole or in part, and is subject to the NIH Public Access Policy. Through acceptance of this federal funding, the NIH has been given a right to make the work publicly available in PubMed Central.

- Georgia Research Alliance (GRA.25.007.EU) to EJS.
- NIH (R01DE026941) to EJS.
- NIH National Cancer Institute (P30CA138292) to SS Ramalingam (for ICD measurements from the Emory Pediatrics/Winship Flow Cytometry Core).

## Supplementary Material

Supplemental data

Supporting data values

## Version 1 05/22/2026

Electronic publication
