# Supplementary material for: Broadening activity of checkpoint blockade agents by intratumoral nucleoside cleavage
Source: JCI Insight. 2026 May 22;11(10):e194142. doi: 10.1172/jci.insight.194142 (PMC13232721; doi:10.1172/jci.insight.194142)
Supplement: Supplemental data [file jciinsight-11-194142-s048.pdf]

Supplemental Figures

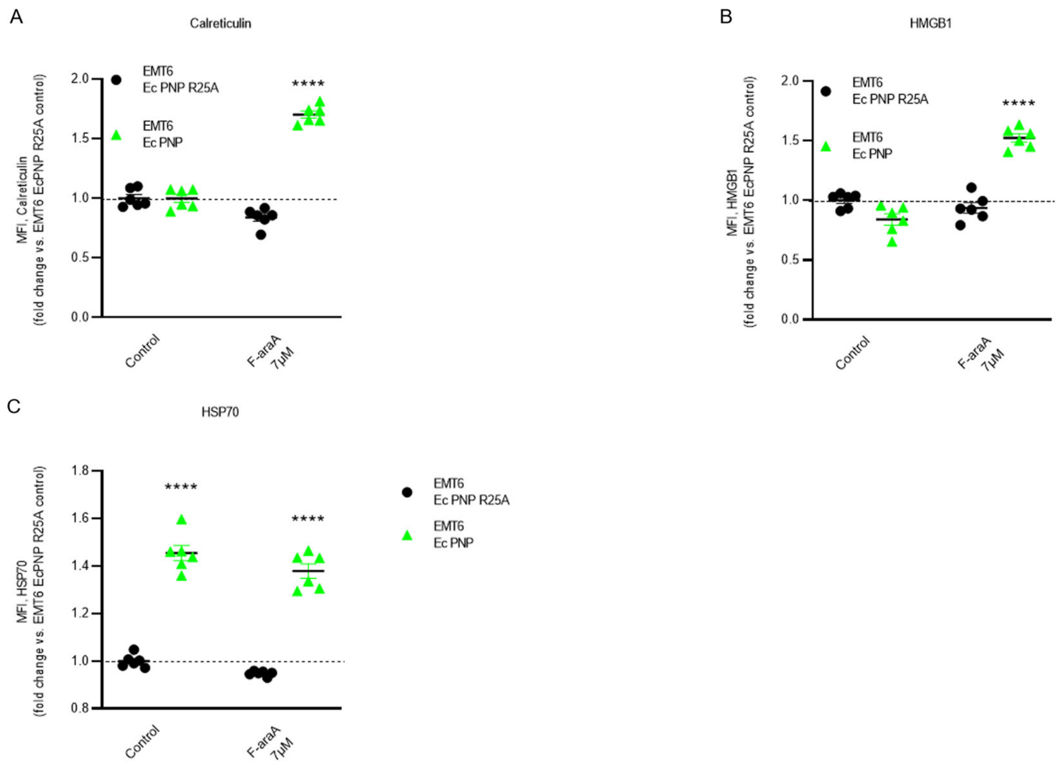


**Supplemental Figure 1**. **Nucleoside prodrug treatment of EMT6 cells expressing mutant (inactive) *E. coli* PNP (R25A).** EMT6 cells encoding wildtype *E. coli* PNP (EcPNP) or the inactive mutant PNP (EcPNP R25A) were treated as in Figure 3B, then fixed, stained for ICD markers, and analyzed by flow cytometry. Cells expressing mutant *E. coli* PNP exhibited basal elevations of HSP70 (i.e., partial ICD phenotype) without nucleoside cleavage, a finding compatible with an unfolded protein response (Zhang *et al., Blood*, 2003). HSP70 baseline values for Ec PNP or Ec PNP R25A were elevated up to 50-100-fold when compared to parental (no PNP) cells (not shown). **** p<0.0001 by two-way ANOVA (post-hoc Tukey comparison).

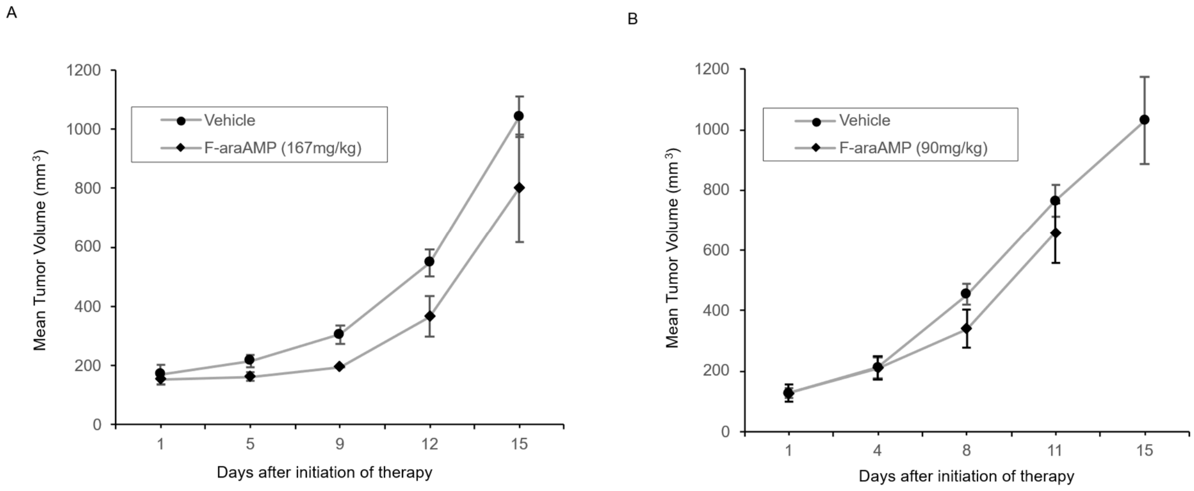


**Supplemental Figure 2. F-araAMP as a single agent (without nucleoside cleavage by PNP) does not confer anti-EMT6 activity.** Mice were implanted with unilateral parental tumors (**A**) or bilateral parental tumors (**B**). F-araAMP was administered IP at a dose of 167 mg/kg or 90 mg/kg on days 1 – 3 after tumors reached a size of 100-200mm$^3$. n = 3-6 mice/condition. Error bars = SEM.

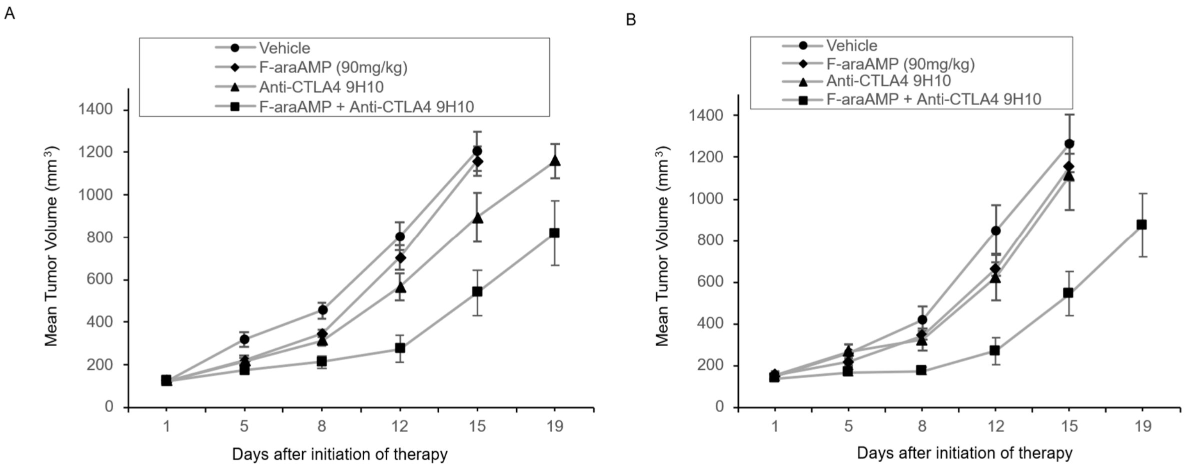


**Supplemental Figure 3. Bilateral EMT6 parental tumors (without *E. coli* PNP) treated with F-araAMP show no anti-cancer activity by the nucleoside and marginal enhancement of ICI activity.** (**A, B**) Experiments are otherwise as shown in Figure 4 except parental tumors were implanted bilaterally. The protocol tested effects of fludarabine phosphate (F-araAMP, 90 mg/kg) with or without anti-CTLA4 on EMT6 tumors that do not express E. coli PNP. Fludarabine phosphate without PNP demonstrated a modest increase of ICI antitumor activity ($p<0.05$, two-tailed Student's t-test) – much smaller than effects elicited by PNP mediated prodrug cleavage (compare to fludarabine phosphate plus ICI treatment shown in Figure 4, B and C). n = 6 mice/condition. Error bars = SEM.

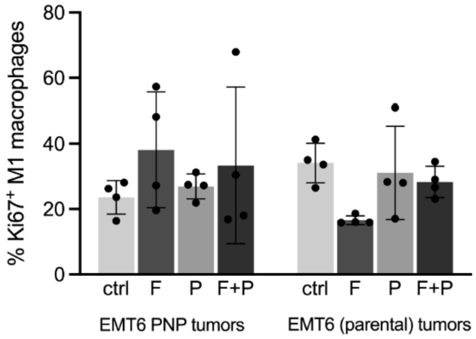


**Supplemental Figure 4. MKI67+ M1-like macrophages are not enriched in tumor parenchyma following treatment with PNP/F-araAMP.** Tumors were established as in Figure 5. Parental EMT6 cells or EMT6 expressing PNP were injected into the right and left flanks of each mouse, respectively, at 5x10^6 cells per flank. Injections with fludarabine phosphate (F), anti-PD-L1 (P), a combination of F-araAMP and anti-PD-L1 (F+P), or vehicle control (ctrl) began on day seven post implantation. Tissues were harvested for analysis of immune cells on day five after start of treatment. One-way ANOVA was used with Tukey's multiple comparison tests for all analyses. No statistically significant differences between groups were observed. n = 4 mice/condition. Error bars = SD.

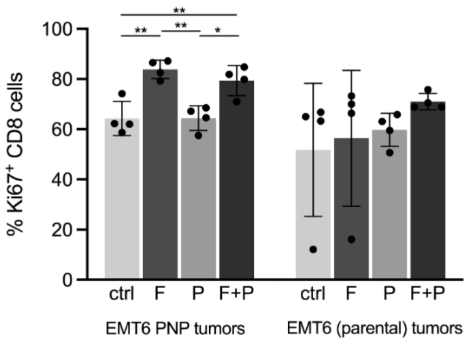


**Supplemental Figure 5. PNP/F-araAMP treatment is associated with increases in % MKI67+ CD8 cells.** Tumors were established as in Figure 5. Parental EMT6 cells, or EMT6 expressing PNP, were injected into the right and left flanks of each mouse, respectively, at 5x10^6 cells per flank. Injections with fludarabine phosphate (F), anti-PD-L1 (P), a combination of F-araAMP and anti-PD-L1 (F+P), or vehicle control (ctrl) began on day seven post implantation. Tissues were harvested for analysis of immune cells on day five after start of treatment. * $p < 0.05$; ** $p < 0.01$. One-way ANOVA was used with Tukey's multiple comparison test for all analyses. n = 4 mice/condition. Error bars = SD.
